# Pharmacological targeting of dopamine D1 or D2 receptors evokes a rapid‐onset parkinsonian motor phenotype in mice

**DOI:** 10.1111/ejn.16622

**Published:** 2024-12-03

**Authors:** Christian del Agua Villa, Mihai Atudorei, Hartwig Roman Siebner, Mattias Rickhag

**Affiliations:** ^1^ Danish Research Centre for Magnetic Resonance (DRCMR), Department of Radiology and Nuclear Medicine Copenhagen University Hospital ‐ Amager and Hvidovre Hvidovre Denmark; ^2^ Department of Neurology Copenhagen University Hospital Bispebjerg and Frederiksberg Copenhagen Denmark; ^3^ Department of Clinical Medicine, Faculty of Health and Medical Sciences University of Copenhagen Copenhagen Denmark; ^4^ Molecular Neuropharmacology and Genetics Laboratory, Department of Neuroscience, Faculty of Health and Medical Sciences University of Copenhagen Copenhagen Denmark

**Keywords:** animal models, dopamine receptor antagonist, open‐field behaviour, parkinsonism

## Abstract

Dopaminergic nigrostriatal denervation in Parkinson's disease (PD) disrupts the functional balance between striatal projecting neurons, leading to aberrant activity in the cortico‐basal ganglia circuit and characteristic motor symptoms. While genetic and toxin‐based animal models are commonly used to mimic PD pathology and behaviour, they have limitations when combined with circuit manipulation tools. This highlights the need for complementary approaches, particularly when combined with viral‐based circuit targeting of specific neuronal subpopulations involved in PD circuit dysfunction. Here, we pursue a pharmacological approach targeting dopamine D1 or D2 receptors to induce dopamine deprivation and to replicate key motor symptoms in PD. We demonstrate a clear dose‐dependent induction of parkinsonian motor behaviour by both a dopamine D1 receptor antagonist (SCH23390) and a D2 receptor antagonist (haloperidol). The motor phenotype is evaluated by considering relevant motor metrics in an open‐field maze platform. The proposed parkinsonian pharmacological model constitutes an acute, flexible approach, which allows parallel brain circuit manipulations.

## Graphical abstract

In this study, we pursue a pharmacological approach targeting dopamine D1 or D2 receptors to induce dopamine deprivation and to replicate key motor symptoms in Parkinson's disease. We demonstrate a clear dose‐dependent induction of parkinsonian motor behaviour by both a D1 receptor antagonist (SCH23390) and a D2 receptor antagonist (haloperidol). The proposed pharmacological model constitutes a feasible approach that allows combining the transient modelling of parkinsonian motor features with fine circuit manipulation approaches.

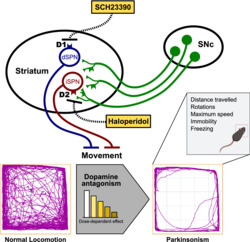

**Abbreviations**
ANOVA: Analysis of Variance
BAC: Bacterial Artificial Chromosome
Cre: Cre recombinase
D1R/D2R: Dopamine D1/D2 receptor
dSPNs/iSPNs: Direct/indirect spiny projection neurons
GABA: Gamma Aminobutyric acid
GENSAT: Gene Expression Nervous System Atlas
L‐DOPA: Levodopa/l‐3,4‐dihydroxyphenylalanine
MAO‐B: Monoamine oxidase B
PD: Parkinson's disease
SEM: Standard error of the mean
TOI: Time of interest
WT: Wild‐type
6‐OHDA: 6‐hydroxydopamine

## 1 INTRODUCTION

Parkinson's disease (PD) is a common, progressive motor syndrome affecting 0.3% of the population (Balestrino & Schapira, 2020). In PD, the degeneration of midbrain dopaminergic nigrostriatal neurons (Volpicelli‐Daley et al., 2011) leads to progressive dopamine depletion in the striatum (Jeong et al., 2023), causing the cardinal motor deficits, often referred to as parkinsonism (tremor, rigidity and brady−/hypokinesia) (Hayes, 2019; McGregor & Nelson, 2019). Animal studies have used different strategies to recreate characteristic parkinsonian pathophysiology, such as genetic, toxin or pharmacological strategies. The selected animal models must be tailored to the specific biological question and must thereby recreate relevant pathophysiological characteristics (Betarbet et al., 2002; Chesselet & Richter, 2011; Dawson et al., 2018). Despite the successful implementation of various models in PD research, such as genetic‐, toxin‐ and drug‐based models, no model is able to holistically represent all facets of the disease (Betarbet et al., 2002; Chesselet & Richter, 2011; Gubellini & Kachidian, 2015; Waku et al., 2021).

The main type of toxin‐based models uses the 6‐hydroxydopamine (6‐OHDA) neurotoxin to elicit adult‐onset parkinsonism, causing severe denervation of dopaminergic striatal neurons (Alcacer et al., 2017; Guimarães et al., 2021). 6‐OHDA models present a high construct validity, as they reliably reproduce nigrostriatal neurodegeneration and striatal dopamine depletion at the cost of extensive histological verification. However, they cannot emulate α‐synuclein aggregation or LB formation (Chia et al., 2020; Lundblad et al., 2004). Predictive validity stands high, as normal motor output can be recovered through the administration of the dopamine precursor L‐DOPA (Alcacer et al., 2017; Andreoli et al., 2021; Lanza et al., 2018). Regarding face validity, 6‐OHDA models highly resemble typical characteristics of the parkinsonian motor phenotype (Andreoli et al., 2021; Chia et al., 2020).

Genetic models emulate familial monogenic forms of PD, causing early neuronal dysfunction and producing diverse parkinsonian phenotypes (Betarbet et al., 2002; Chesselet & Richter, 2011; Good et al., 2011). Their construct validity varies with different models showing a mix of motor impairments, α‐synuclein aggregation and striatal dopamine depletion. Their predictive and face validity models are often limited for motor symptoms, and the research focus is usually on cellular dysfunction seen in PD (Chesselet & Richter, 2011; Chia et al., 2020). In addition, breeding specific mouse strains for genetic models can restrict the use of other genetic tools, such as cre‐driver lines. These limitations highlight the need for complementary approaches to toxin‐ and genetic‐based models.

Striatal dopamine depletion evokes an imbalance between the two major projection neurons in the striatum: direct‐pathway medium spiny neurons (dSPNs) and indirect‐pathway medium spiny neurons (iSPNs) (McGregor & Nelson, 2019). According to the classical model, the activation of dSPNs induces an overall decrease in basal ganglia inhibitory output, disinhibiting the thalamus and promoting movement (Albin et al., 1989; Bolam et al., 2000; Lanciego et al., 2012; McGregor & Nelson, 2019). On the other hand, the activation of iSPNs increases basal ganglia output, inhibiting the thalamo‐cortical circuits and suppressing movement (Albin et al., 1989; Bolam et al., 2000; Lanciego et al., 2012; McGregor & Nelson, 2019). iSPNs and dSPNs display distinct dopamine receptor expression patterns. Dopamine D1 receptors (D1R) are mainly expressed by dSPNs, while dopamine D2 receptors (D2R) are expressed by iSPNs (Bateup et al., 2010; Deng et al., 2006; McGregor & Nelson, 2019). Following the classical model of basal ganglia circuitry, dopamine has a bivalent effect on striatal locomotor output, depending on the targeted neuronal subset (McGregor & Nelson, 2019). However, recent work advocates that both striatal pathways show concurrent activation during movement to facilitate selected actions and inhibit competing ones (Cui et al., 2013; Mink, 1996, 2003). Dopamine D2 receptors present a complex expression distribution not limited to iSPNs as they can be found on presynaptic GABAergic and glutamatergic synaptic terminals, thereby regulating the release probability of the respective neurotransmitter. Consequently, D2R activation orchestrates complex alterations in the striatal circuit activation patterns from both presynaptic and postsynaptic ends, all influencing the striatal output (Sippy & Tritsch, 2023).

Drug‐based models exploit the role of dopamine signalling in the striatum. By using D1R and D2R, the balance between the direct and indirect pathways can be altered, which in turn modulates motor output, potentially leading to hyperlocomotion or catalepsy (De Ryck et al., 1980; Morelli & Di Chiara, 1985; Undie & Friedman, 1988). Pharmacological models have been utilized both in vivo and ex vivo to answer different questions of PD pathophysiology (Andreoli et al., 2021; Avila‐Luna et al., 2018; Bateup et al., 2010; Maltese et al., 2021; Masini & Kiehn, 2022; Scheel‐Krüger & Magelund, 1981).

Here, we systematically investigate the motor effects of varying doses of dopamine D1R or D2R antagonists in mice in a previously underutilized platform, the open‐field test. Although pharmacological models of PD have been used for many years, the field lacks comprehensive studies analysing motor phenotypes across a broad dose range in an open‐field arena. We hypothesized that acute administration of the dopamine D1 receptor antagonist (SCH23390) or D2 receptor antagonist (haloperidol) produces similar transient dose‐dependent effects on spontaneous horizontal whole‐body movements in the open‐field maze, mimicking a parkinsonian motor phenotype. Following our investigation, we would recommend a dose of 0.02 mg/kg of SCH23390, while for haloperidol we would recommend doses between 0.2 and 0.5 mg/kg.

## 2 MATERIALS AND METHODS

### 2.1 Animals

A mouse strain with C57BL/6 background (Chrna2‐Cre) was chosen to establish the pharmacological modelling of motor output. This strain was generated through the GENSAT project, mouse strain Chrna2‐Cre (B6.FVB (Cg)‐Tg (Chrna2‐cre).OE25Gsat/Mmucd, stock number 0376 54‐UCD). Only Cre‐/WT individuals were used for pharmacological modulation. Male and female Chrna2‐Cre mice were sourced from the Mutant Mouse Regional Resource Centre, UC Davis, California and were preserved in a hemizygous state by backcross breeding with C57B1/6 N mice to counteract gene disruption concerns that BAC stochastic insertion could cause. For the experimental setup, n = 48 mice were used (38 females and 10 males). The mice were group‐housed separately under a 12‐hour light/dark cycle, with ad libitum food and water. The animals used in this study were more than 16 weeks old, with weights ranging from 22 to 35 g. All experiments were carried out in compliance with the standards of the Danish Animal Experimentation Inspectorate (animal license number: 2021‐15‐0201‐01056) under the supervision of a local animal welfare committee. Good efforts were made to minimize animal suffering and the overall number of mice used in the study.

### 2.2 Pharmacological compounds

SCH23390 ((*R*)‐(+)‐Scheme 23390 hydrochloride, D054) was sourced from Merck/Sigma‐Aldrich (Burlington, MA, USA) and diluted into distilled water to create stock solutions of 1 mg/ml. Subsequent dilutions to target doses (0.005 mg/kg, 0.01 mg/kg, 0.02 mg/kg, 0.05 mg/kg and 0.5 mg/kg for SCH23390) (Hjorth & Carlsson, 1988; Morelli & Di Chiara, 1985) were prepared using saline solution. Haloperidol (HalDol®/Serenase®) was sourced from Janssen Pharmaceuticals (Beerse, Belgium) in an aqueous solution. A saline solution was used for the preparation of the diluted injectable doses (0.05 mg/kg, 0.1 mg/kg, 0.2 mg/kg, 0.5 mg/kg and 1 mg/kg) (Waku et al., 2021). Intraperitoneal injections were performed immediately before behavioural trials (SCH23390, haloperidol or saline). We allowed at least five days between different drug injections to allow proper metabolization and excretion (washout period). A total of four maximum injections and behavioural assessments for each animal's experimental use.

### 2.3 Study design

The animals were divided into groups of four for simultaneous testing and assigned to different treatments in a lattice design. One of the animals in each open‐field test was assigned to the saline group, the rest being assigned to the different treatment doses. For every behavioural assessment, a different animal of the group will be assigned to the saline group. This was done for platform validation purposes as well as reducing inter‐subject variability when comparing experimental groups to the control. The doses of SCH23390 and haloperidol were randomly assigned. No animals received the same combination of drug and dose. Therefore, this should be considered as an acute administration of dopamine antagonists. Quadrant equivalence platform validation (differences in locomotion between quadrants), and successive open‐field exposure assessments (effect of consecutive open‐field testing in habituation) were performed inside the saline group. Quadrant placement did not significantly influence motor metrics, while successive open‐field exposure did influence motor metrics. Early open‐field testing rounds presented a higher locomotor output than later rounds (habituation). The design considered these potential confounding effects; as groups had comparable average open‐field test round and quadrant distribution, therefore confirming that these confounding factors did not have an effect on motor measurements. Animals were randomly distributed among quadrants, with no bias of saline group towards a specific quadrant. There were no statistical differences in the average open‐field test number between groups.

### 2.4 Open‐field behaviour test

We assessed spontaneous motor output and locomotion in an open‐field platform consisting of a large, square arena (white background, black walls) subdivided into four equally sized quadrants of dimensions 50x50x35cm. A USB 2.0 CMOS Camera (Stoelting Co., IL, USA) raised one meter above the centre of the maze and simultaneously recorded all quadrants. The AnyMaze 7 Video Tracking Software (Stoelting Co., IL, USA) performed the movement tracking. This software allows simultaneous detection of the head, tail and body centre. The distance travelled and number of mobile episodes were obtained using the default settings of the tracking engine, while the freezing and immobility thresholds were set at two seconds (2 s). We discarded maximum speed recordings over 0.8 m/s as they are likely to represent occasional one‐frame jittering tracking issues. The tracking was performed under constant artificial lighting conditions with white noise to cover any unwanted environmental disturbances. All behavioural tests were conducted within two clearly defined starting timeslots of 30 minutes, starting either at 9 am or at noon. The paradigm used to test dopamine antagonists SCH23390 and haloperidol lasted for 90 minutes after intraperitoneal (IP) injection without habituation (total time *=* 90 min).

### 2.5 Motor output metrics

Several motor output metrics were used to evaluate different locomotor characteristics of the animals in the open field. Distance travelled is a widely used metric in open‐field studies for its tracking ease and for its ability to measure overall locomotion and exploratory behaviour (Alcacer et al., 2017; Avila‐Luna et al., 2018; Hjorth & Carlsson, 1988; Löschmann et al., 1992; Masini & Kiehn, 2022; Schulz et al., 1985).

Rotations are defined as the number of 360° turns and can reveal bias towards unilateral movements. Maximum speed, measured as the absolute highest speed reached during a five‐minute interval, was chosen to explore the feasibility of assessing movement amplitude and bradykinesia in an open‐field setup and to evaluate whether locomotor bouts speed was affected by dopamine antagonism.

Immobility was defined as a lack of transitory movement where the centre of the animal remains stationary while allowing head and tail movements. Mobile episodes ‐ the number of times in which the animals initiate transitory movement, were chosen to provide a representation of movement initiation akin to parkinsonism. The combined length of these episodes constituted the total percentage of time immobile. The minimum immobility time threshold was set at two seconds (2 s).

Freezing behaviour was defined as a completely still, catatonic behaviour, where head and tail movements are absent. This metric is stricter than immobility but portrays important similar parkinsonian features such as movement initiation. The time freezing is always a subset of the time immobile, as the base exclusion criteria (lack of transitory movement) is shared while freezing additionally excludes stationary movements. Freezing metrics allow the measuring of fine akinetic states, representing potential movement initiation impairments. Freezing can, however, represent an anxiety response in rodents. The combined length of these episodes constituted the total percentage of time freezing. The minimum freezing time threshold was set at two seconds (2 s).

### 2.6 Statistical analysis

To evaluate the statistical differences of dopamine antagonist to the saline control in different metrics over time we used a two‐way analysis of variance (ANOVA), with Sidak post‐hoc analysis. We chose a pharmacological active period for further analysis of the motor output comprised between 10‐ and 60 minutes post‐injection. The total distance travelled within the timeframe, number of rotations, proportion of time freezing and time immobile, number of freezing episodes and mobile episodes and maximum speed achieved during the specified period were analysed using fixed‐effect one‐way ANOVA, followed by a Dunnett's multiple comparisons of the means (column graphs). All results are depicted as mean ± standard error of the mean (SEM). A significance level of p < 0.05 was chosen for all comparisons. The statistical analysis was performed using GraphPad Prism 9.5 (GraphPad Software LLC, Boston, MA).

## 3 RESULTS

### 3.1 Haloperidol and SCH23390 effectively diminish total distance travelled and overall locomotor activity

**FIGURE 1:**
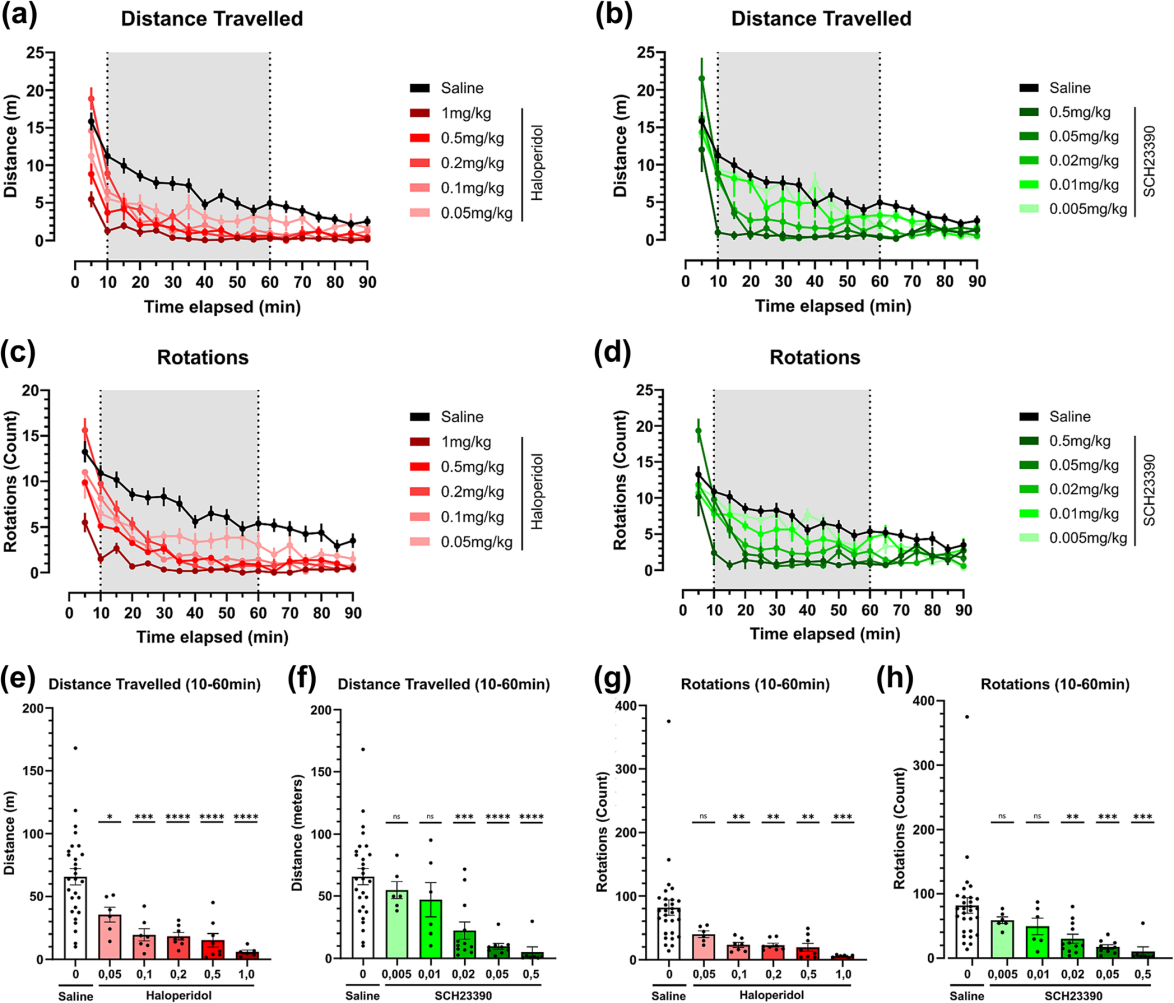
Distance travelled and rotational behaviour locomotor metrics of dopamine D2 receptor antagonist haloperidol and D1 receptor antagonist SCH23390 in an open‐field test across an array of doses. (a‐b) Distance travelled over time in five‐minute intervals of dopamine antagonist haloperidol (a) (doses of 1 mg/kg (n = 6), 0.5 mg/kg (n = 8), 0.2 mg/kg (n = 8), 0.1 mg/kg (n = 7) and 0.05 mg/kg (n = 6)) and SCH23390 (b) (doses of 0.5 mg/kg (n = 7), 0.05 mg/kg (n = 9), 0.02 mg/kg (n = 12), 0.01 mg/kg (n = 6) and 0.005 mg/kg (n = 6)) compared against a saline control (n = 28). (c‐d) Rotations over time in five‐minute intervals of the selected doses of haloperidol (c) and SCH23390 (d) compared against a saline control. (e‐f) Total distance travelled within the timeframe of interest (TOI, 10 to 60 minutes) for the entire dosing array of haloperidol (e) (F [5, 57] = 11.69) and SCH23390 (f) (F [5, 62] = 10.80). (g‐h) Total number of rotations within the timeframe of interest (TOI, 10 to 60 minutes) for the entire dosing array of haloperidol (g) (F [5, 58] = 5.641) and SCH23390 (h) (F [5, 63] = 5.117). Brackets above the column graphs denote a one‐way ANOVA analysis, followed by Dunnett's multiple pairwise comparison of the means. NS indicates *p* > 0.05, * *p* < 0.05; ** *p* < 0.01; *** *p* < 0.001; **** *p* < 0.0001. Error bars represent ±SEM.

To elicit a hypokinetic motor phenotype mimicking parkinsonism and to delineate the compounds efficacy, we performed a dose–response study of high‐affinity dopamine D1 (SCH22390) and D2 receptor antagonists (haloperidol). SCH23390 and haloperidol effects on overall locomotor output were tested through a five‐dose range of each compound (0.5 mg/kg, 0.05 mg/kg, 0.02 mg/kg, 0.01 mg/kg and 0.005 mg/kg for SCH23390; 1.0 mg/kg, 0.5 mg/kg, 0.2 mg/kg, 0.1 mg/kg and 0.05 mg/kg for haloperidol) and compared to a saline‐injected group. Two distinct motor metrics were chosen to describe overall locomotor output: distance travelled and rotations (Figure 1). Distance travelled decreased over time as the animals in the saline group familiarized themselves with the environment (Figure 1a‐b). Distance travelled decreases over time and is intensified with increasingly high dopamine receptor antagonist doses (Figure 1a‐b). We compared the distance travelled change over time of saline and different doses using a 2‐way ANOVA. The p‐value heatmap plot (Tables 1 & 2) justifies establishing a timeframe of interest (TOI) comprised between drawn between 10‐ and 60 minutes post‐injection to study the drug's maximum effect according to their established pharmacodynamic profile (Froemming et al., 1989; Undie & Friedman, 1988). We compared the distance travelled inside the TOI using a one‐way ANOVA, resulting in statistical differences along the entire haloperidol dose range (Figure 1e), while only doses equal to and above 0.02 mg/kg SCH23390 showed statistical significance (Figure 1f). Both compounds showed clear dose‐dependency (Figure 1e‐f).

**TABLE 1:** Heatmaps for two‐way ANOVAs throughout the entire experimental timeline (0–90 minutes) with Sidak's post‐hoc analysis of p‐value matrices with regards to the motor metrics of (top to bottom): distance travelled, maximum speed, mobile episodes, time immobile (% of time), freezing episodes and time spent freezing (% of time) after the dopamine D2R antagonist haloperidol administration at all experimental concentrations.

Distance travelled																		
	5	10	15	20	25	30	35	40	45	50	55	60	65	70	75	80	85	90
**1 mg/kg**	0.0005	0.0001	0.0001	0.0001	0.0001	0.0001	0.0001	0.0001	0.0001	0.0016	0.0049	0.0017	0.0028	0.0094	0.0611	0.0598	0.113	0.1545
**0.5 mg/kg**	0.2165	0.0652	0.7306	0.0017	0.0526	0.0027	0.0134	0.0167	0.0419	0.0022	0.0606	0.023	0.0016	0.139	0.9999	0.8273	0.9999	0.6881
**0.2 mg/kg**	0.9999	0.9999	0.2749	0.0363	0.0077	0.5072	0.0158	0.1022	0.0012	0.045	0.0173	0.0022	0.0106	0.9801	0.9999	0.9385	0.3821	0.5576
**0.1 mg/kg**	0.9999	0.2189	0.5634	0.0004	0.024	0.0006	0.0151	0.9442	0.0741	0.0104	0.8659	0.035	0.0632	0.717	0.1354	0.7709	0.9965	0.9999
**0.05 mg/kg**	0.9894	0.5755	0.2317	0.9989	0.9203	0.2743	0.9999	0.9999	0.9209	0.9999	0.9999	0.9999	0.9999	0.9999	0.9989	0.9999	0.9999	0.9999

Maximum speed																		
	5	10	15	20	25	30	35	40	45	50	55	60	65	70	75	80	85	90
**1 mg/kg**	0.9999	0.9999	0.9999	0.9999	0.9999	0.9999	0.2771	0.1618	0.0237	0.697	0.0081	0.0801	0.606	0.538	0.9999	0.1598	0.0011	0.9834
**0.5 mg/kg**	0.9999	0.8094	0.9999	0.7635	0.9999	0.5413	0.0695	0.9916	0.9999	0.1473	0.6007	0.5104	0.0001	0.99	0.9999	0.9643	0.9999	0.9939
**0.2 mg/kg**	0.9999	0.9999	0.9999	0.9999	0.9999	0.9999	0.9999	0.9995	0.8311	0.9744	0.9999	0.5891	0.7757	0.9993	0.9999	0.8234	0.9999	0.9888
**0.1 mg/kg**	0.9999	0.9999	0.9999	0.9999	0.9999	0.9826	0.9978	0.9999	0.9715	0.125	0.9999	0.9999	0.2304	0.9663	0.7685	0.9999	0.9999	0.9999
**0.05 mg/kg**	0.9999	0.9999	0.9999	0.9999	0.9999	0.9999	0.9999	0.9999	0.9999	0.9999	0.9999	0.9999	0.9999	0.9999	0.9999	0.9999	0.9999	0.9999

Mobile episodes																		
	5	10	15	20	25	30	35	40	45	50	55	60	65	70	75	80	85	90
**1 mg/kg**	0.5608	0.0001	0.0003	0.0001	0.0001	0.0001	0.0001	0.0001	0.0001	0.0001	0.0001	0.0001	0.0001	0.007	0.051	0.1598	0.1825	0.7137
**0.5 mg/kg**	0.9999	0.9999	0.9999	0.6214	0.9999	0.949	0.1577	0.004	0.7617	0.0001	0.9994	0.1086	0.0001	0.5208	0.9999	0.4883	0.9999	0.9752
**0.2 mg/kg**	0.9999	0.9999	0.9999	0.9638	0.9089	0.8825	0.2569	0.0029	0.0002	0.0098	0.0642	0.0004	0.0039	0.9637	0.9999	0.9997	0.4447	0.6213
**0.1 mg/kg**	0.9999	0.9999	0.9999	0.9999	0.9999	0.9217	0.9941	0.9999	0.9938	0.8289	0.9999	0.0304	0.9978	0.9757	0.0244	0.9999	0.9999	0.9999
**0.05 mg/kg**	0.9999	0.9999	0.9999	0.9999	0.9999	0.9999	0.9999	0.9999	0.9999	0.9999	0.9999	0.9999	0.9999	0.9999	0.9999	0.9999	0.9999	0.9999

Time immobile (%)																		
	5	10	15	20	25	30	35	40	45	50	55	60	65	70	75	80	85	90
**1 mg/kg**	0.0003	0.0001	0.0001	0.0001	0.0001	0.0001	0.0001	0.0001	0.0001	0.0002	0.0057	0.0008	0.0028	0.0051	0.0423	0.0622	0.1594	0.1839
**0.5 mg/kg**	0.7509	0.3338	0.9986	0.0096	0.1468	0.0073	0.0356	0.0116	0.0128	0.0003	0.1469	0.0068	0.0014	0.1017	0.9966	0.9457	0.9999	0.9204
**0.2 mg/kg**	0.9999	0.9881	0.4453	0.0564	0.0182	0.0496	0.0035	0.0039	0.0001	0.0018	0.0192	0.0016	0.008	0.9996	0.9981	0.6355	0.4652	0.5198
**0.1 mg/kg**	0.9999	0.7686	0.3008	0.0003	0.0284	0.0062	0.1383	0.9277	0.197	0.0203	0.9999	0.013	0.3715	0.9215	0.185	0.9758	0.9998	0.9999
**0.05 mg/kg**	0.9999	0.9174	0.5135	0.9999	0.9999	0.605	0.9999	0.9999	0.9999	0.9999	0.9999	0.9999	0.9999	0.9999	0.9997	0.9999	0.9999	0.9999

Freezing episodes																		
	5	10	15	20	25	30	35	40	45	50	55	60	65	70	75	80	85	90
**1 mg/kg**	0.8258	0.9999	0.9999	0.9999	0.9999	0.9303	0.7308	0.0054	0.0001	0.0002	0.0001	0.0001	0.0001	0.0158	0.009	0.5218	0.0067	0.9999
**0.5 mg/kg**	0.9999	0.9999	0.9999	0.9999	0.9999	0.9979	0.9999	0.9999	0.9999	0.9999	0.9142	0.033	0.0001	0.0565	0.9999	0.9999	0.6408	0.9999
**0.2 mg/kg**	0.9999	0.9999	0.9999	0.9812	0.9999	0.9999	0.9999	0.9999	0.9999	0.9999	0.9999	0.9999	0.9999	0.9999	0.9999	0.9999	0.9999	0.9999
**0.1 mg/kg**	0.9999	0.4065	0.2383	0.0142	0.0343	0.9999	0.9999	0.9999	0.9999	0.9999	0.9999	0.9999	0.9999	0.9999	0.9999	0.9999	0.9999	0.9999
**0.05 mg/kg**	0.9999	0.9277	0.0711	0.7622	0.2122	0.3468	0.8873	0.994	0.9995	0.9999	0.9189	0.9999	0.9999	0.9999	0.9999	0.9999	0.9999	0.9999

Time freezing (%)																		
	5	10	15	20	25	30	35	40	45	50	55	60	65	70	75	80	85	90
**1 mg/kg**	0.786	0.0087	0.0001	0.0001	0.0001	0.0001	0.0001	0.0001	0.0001	0.0001	0.0001	0.0001	0.0001	0.0001	0.0002	0.0002	0.0015	0.0263
**0.5 mg/kg**	0.9999	0.9999	0.9976	0.9605	0.4832	0.8424	0.0873	0.0218	0.7445	0.0001	0.3616	0.0017	0.0001	0.038	0.9999	0.9935	0.9999	0.9973
**0.2 mg/kg**	0.9838	0.9999	0.9999	0.9999	0.8239	0.1251	0.0243	0.0013	0.0012	0.0243	0.0137	0.0001	0.0046	0.9196	0.9999	0.7891	0.0271	0.3644
**0.1 mg/kg**	0.9999	0.9999	0.9997	0.7565	0.5483	0.6198	0.528	0.9999	0.7291	0.2844	0.9323	0.2584	0.59	0.5282	0.0225	0.9818	0.8734	0.9999
**0.05 mg/kg**	0.9999	0.9999	0.9999	0.9999	0.2816	0.9995	0.9999	0.9999	0.9999	0.9999	0.9999	0.9999	0.9999	0.9999	0.9999	0.9999	0.9999	0.9999

**TABLE 2:** Heatmaps for two‐way ANOVAs throughout the entire experimental timeline (0–90 minutes) with Sidak's post‐hoc analysis of p‐value matrices with regards to the motor metrics of (top to bottom): distance travelled, maximum speed, mobile episodes, time immobile (% of time), freezing episodes and time spent freezing (% of time) after the dopamine D1R antagonist SCH23390 administration at all experimental concentrations.

Distance travelled																		
	5	10	15	20	25	30	35	40	45	50	55	60	65	70	75	80	85	90
**0.5 mg/kg**	0.9999	0.0001	0.0001	0.0005	0.0001	0.0002	0.0001	0.0002	0.0005	0.0027	0.3904	0.0015	0.0026	0.5146	0.9999	0.9999	0.9999	0.9999
**0.05 mg/kg**	0.9999	0.9999	0.0046	0.0001	0.0636	0.0001	0.0001	0.0002	0.001	0.3247	0.1179	0.0055	0.0035	0.4915	0.9999	0.9999	0.9999	0.9999
**0.02 mg/kg**	0.9999	0.9999	0.0912	0.015	0.2902	0.3738	0.0088	0.9459	0.0144	0.9993	0.4944	0.8056	0.0703	0.0243	0.709	0.9999	0.9999	0.7127
**0.01 mg/kg**	0.9999	0.9999	0.9999	0.9999	0.9938	0.9999	0.9999	0.9999	0.9999	0.9999	0.9999	0.9999	0.9999	0.9999	0.9999	0.9999	0.9807	0.9999
**0.005 mg/kg**	0.9999	0.9999	0.9999	0.9999	0.9999	0.9999	0.9999	0.9999	0.9999	0.9999	0.9999	0.6624	0.9999	0.9999	0.9999	0.9088	0.9967	0.4957

Maximum speed																		
	5	10	15	20	25	30	35	40	45	50	55	60	65	70	75	80	85	90
**0.5 mg/kg**	0.9999	0.1367	0.0909	0.126	0.0164	0.0203	0.0001	0.0978	0.0076	0.2615	0.0003	0.0818	0.0001	0.6531	0.9999	0.715	0.9983	0.9999
**0.05 mg/kg**	0.9999	0.9999	0.997	0.0011	0.1597	0.0002	0.0001	0.048	0.0005	0.7852	0.4554	0.0301	0.0775	0.4311	0.9999	0.9999	0.9999	0.9999
**0.02 mg/kg**	0.9957	0.9999	0.9997	0.857	0.9823	0.2705	0.2731	0.5103	0.9993	0.9999	0.9999	0.9999	0.9999	0.3621	0.9999	0.9999	0.9999	0.9999
**0.01 mg/kg**	0.9999	0.9999	0.9999	0.9999	0.9999	0.9999	0.9999	0.9999	0.9999	0.9999	0.9999	0.9999	0.9999	0.9999	0.9999	0.9993	0.9999	0.9999
**0.005 mg/kg**	0.9999	0.9999	0.9999	0.9999	0.9999	0.9999	0.9999	0.7753	0.9999	0.9999	0.9999	0.9999	0.9999	0.9999	0.9999	0.9999	0.9999	0.8091

Mobile episodes																		
	5	10	15	20	25	30	35	40	45	50	55	60	65	70	75	80	85	90
**0.5 mg/kg**	0.9999	0.6505	0.2364	0.2121	0.0014	0.1686	0.0634	0.0176	0.1679	0.1146	0.9964	0.0014	0.2307	0.9999	0.9999	0.9999	0.9999	0.9999
**0.05 mg/kg**	0.9999	0.9999	0.9999	0.0001	0.0921	0.0001	0.0001	0.026	0.0241	0.5191	0.9999	0.0114	0.0002	0.985	0.9999	0.9999	0.9999	0.9999
**0.02 mg/kg**	0.9985	0.9999	0.9999	0.5178	0.5135	0.2431	0.2725	0.626	0.1382	0.9999	0.9999	0.9999	0.1995	0.2914	0.9974	0.9999	0.9999	0.9817
**0.01 mg/kg**	0.9999	0.9917	0.9999	0.9999	0.9999	0.9999	0.9999	0.9999	0.9999	0.9999	0.9999	0.9999	0.9999	0.9999	0.9999	0.9999	0.9999	0.9999
**0.005 mg/kg**	0.9999	0.9999	0.9994	0.9999	0.9999	0.9999	0.9999	0.9999	0.9999	0.9999	0.9999	0.9999	0.9999	0.9999	0.9999	0.9999	0.9999	0.8491

Time immobile (%)																		
	5	10	15	20	25	30	35	40	45	50	55	60	65	70	75	80	85	90
**0.5 mg/kg**	0.9999	0.0001	0.0001	0.0028	0.0001	0.0003	0.0001	0.0003	0.001	0.0028	0.9933	0.0017	0.0026	0.9998	0.9999	0.9999	0.9999	0.9999
**0.05 mg/kg**	0.9999	0.9999	0.0061	0.0001	0.0806	0.0001	0.0001	0.0005	0.0008	0.7196	0.229	0.0045	0.0038	0.288	0.9999	0.9999	0.9999	0.9999
**0.02 mg/kg**	0.9999	0.9999	0.1455	0.0571	0.5956	0.7001	0.0094	0.9928	0.0236	0.9999	0.8612	0.9564	0.0643	0.0334	0.342	0.9999	0.9999	0.6521
**0.01 mg/kg**	0.9999	0.9999	0.9999	0.9999	0.9999	0.9999	0.9999	0.9999	0.9999	0.9999	0.9999	0.9999	0.9999	0.9999	0.9999	0.9999	0.9999	0.9999
**0.005 mg/kg**	0.9999	0.9999	0.9999	0.9999	0.9999	0.9999	0.9999	0.9999	0.9999	0.9999	0.9999	0.9002	0.9999	0.9999	0.9999	0.9632	0.9998	0.8703

Freezing episodes																		
	5	10	15	20	25	30	35	40	45	50	55	60	65	70	75	80	85	90
**0.5 mg/kg**	0.9999	0.9999	0.9999	0.9999	0.9999	0.9999	0.9999	0.9999	0.9999	0.9999	0.01	0.9999	0.4046	0.9995	0.9999	0.9999	0.9999	0.9999
**0.05 mg/kg**	0.9902	0.9999	0.8815	0.9006	0.9999	0.9996	0.9999	0.3749	0.4993	0.9999	0.9999	0.9999	0.0005	0.8914	0.9999	0.9999	0.9994	0.9999
**0.02 mg/kg**	0.9999	0.9999	0.9905	0.9999	0.9999	0.9999	0.9999	0.9999	0.9999	0.9999	0.9999	0.9999	0.9999	0.9999	0.9808	0.9999	0.9999	0.9999
**0.01 mg/kg**	0.9999	0.9999	0.9999	0.9999	0.9999	0.9999	0.9999	0.9999	0.9999	0.9999	0.9999	0.9999	0.5555	0.9999	0.9999	0.9999	0.9999	0.9999
**0.005 mg/kg**	0.9999	0.9999	0.9999	0.9999	0.9999	0.9999	0.9999	0.9999	0.9999	0.9999	0.9999	0.9716	0.9999	0.9999	0.9999	0.9999	0.9999	0.9999

Time freezing (%)																		
	5	10	15	20	25	30	35	40	45	50	55	60	65	70	75	80	85	90
**0.5 mg/kg**	0.9999	0.1817	0.0545	0.1921	0.2877	0.2514	0.2284	0.1804	0.6055	0.1217	0.9719	0.0021	0.0016	0.9999	0.9999	0.9999	0.9999	0.9999
**0.05 mg/kg**	0.9458	0.9999	0.9999	0.1564	0.1289	0.001	0.0001	0.0001	0.0002	0.9965	0.2513	0.0109	0.0001	0.3966	0.9999	0.9999	0.9999	0.9999
**0.02 mg/kg**	0.9999	0.9999	0.962	0.7791	0.094	0.1789	0.0011	0.058	0.012	0.9176	0.4568	0.4761	0.0003	0.0248	0.2264	0.9999	0.9996	0.8632
**0.01 mg/kg**	0.9999	0.9999	0.9999	0.9999	0.9999	0.9999	0.9999	0.9999	0.9999	0.9999	0.9999	0.9999	0.9999	0.9999	0.9999	0.9999	0.9999	0.9999
**0.005 mg/kg**	0.9999	0.9999	0.9999	0.9999	0.9999	0.9999	0.9999	0.9999	0.9999	0.9999	0.9999	0.9998	0.9999	0.9999	0.9999	0.9999	0.9999	0.9999

The number of rotations decreased over time due to the animal's habituation to the open field (Figure 1c‐d). Similar dose‐dependent decreases compared to the distance travelled were seen in all dose groups through the entire experiment duration (Figure 1c‐d) and the TOI (Figure 1g‐h). No dose‐dependent bias towards clockwise or counterclockwise rotations was found for SCH23390, and only the 1 mg/kg haloperidol dose displayed a significantly increased ratio of clockwise rotations (*data not shown*).

### 3.2 Dopamine receptor antagonism mimic bradykinetic effects resulting in diminished maximum speed

**FIGURE 2:**
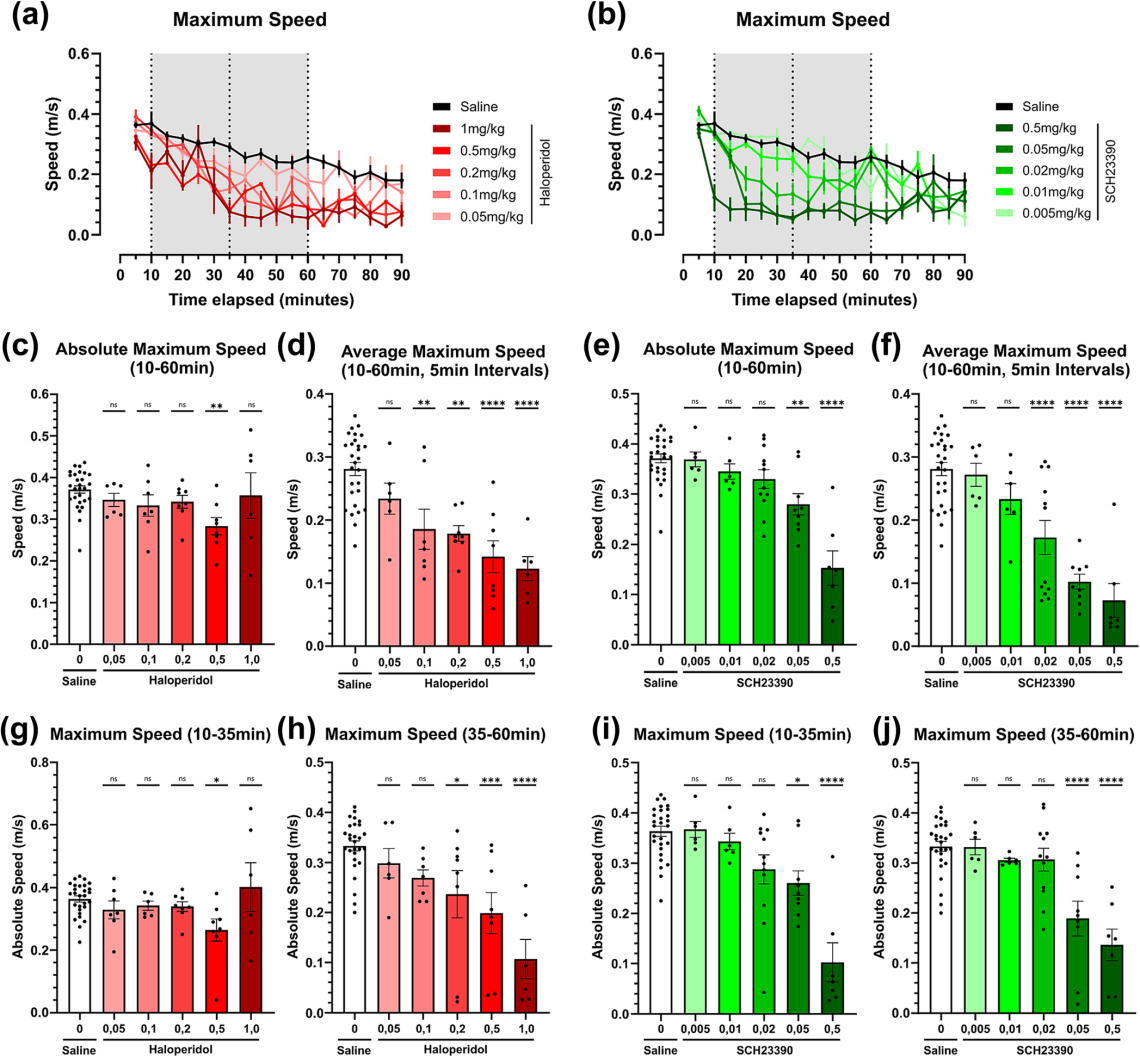
Maximum speed locomotor metrics of dopamine D2 receptor antagonist haloperidol and D1 receptor antagonist SCH23390 in an open‐field test across a wide array of doses. (a) Maximum speed achieved over time in five‐minute intervals of dopamine antagonist haloperidol corresponding to doses of 1 mg/kg (n = 6), 0.5 mg/kg (n = 8), 0.2 mg/kg (n = 8), 0.1 mg/kg (n = 7) and 0.05 mg/kg (n = 6) compared against a saline control (n = 28). (b) Maximum speed achieved over time in five‐minute intervals of dopamine antagonist SCH23390 through the selected doses of 0.5 mg/kg (n = 7), 0.05 mg/kg (n = 9), 0.02 mg/kg (n = 12), 0.01 mg/kg (n = 6) and 0.005 mg/kg (n = 6) compared against a saline control (n = 28). (c‐d) Absolute maximum speed (c) (F [5, 57] = 2.651) and average maximum speed (d) (F [5, 57] = 13.30) achieved within the timeframe of interest (TOI) comprised between 10‐ and 60‐minutes post‐injection for dopamine antagonist haloperidol. (e‐f) Absolute maximum speed (e) (F [5, 62] = 18.83) and average maximum speed (f) (F [5, 62] = 21.09) achieved within the timeframe of interest (TOI) comprised between 10‐ and 60‐minutes post‐injection for the dopamine antagonist SCH23390. Antagonists are compared through the stipulated dose range compared against a saline control. (g‐h) Averaged maximum speeds achieved within 5‐minute intervals of dopamine antagonists' haloperidol (g) and SCH23390 (h) through the stipulated dose range compared against a saline control. (g‐j) Absolute maximum speed achieved within the TOI, split into two timeframes. Haloperidol 10‐ to 35‐minutes (g) (F [5, 57] = 2.505), and 35‐ to 60‐minutes post‐injection (h) (F [5, 57] = 9.810). SCH23390 10 to 35 minutes (i) (F [5, 62] = 17.68), and 35‐ to 60minutes post‐injection (j) (F [5, 62] = 14.35). Antagonists are compared through the stipulated dose range compared against a saline control. Brackets above the column graphs denote a one‐way ANOVA analysis, followed by Dunnett's multiple pairwise comparison of the means. NS indicates *p* > 0.05, * *p* < 0.05; ** *p* < 0.01; *** *p* < 0.001; **** *p* < 0.0001. Error bars represent ±SEM.

Maximum speed showed a steady and linear decrease over time for the saline group (Figure 2a‐b). Both dopamine antagonists presented lower, dose‐dependent, maximum speeds compared to the saline group in the same timeframe. However, the rate of decline in high doses of SCH23390 was sharper compared to high doses of haloperidol (Figure 2a‐b). A two‐way ANOVA with a post‐hoc Sidak analysis of the maximum speed is provided (Tables 1 and 2). We also compared different methods to evaluate maximum speed inside the TOI, both absolute maximum speed during the 50‐minute‐long interval (absolute maximum speed) (Figure 2c,e) and averaging the maximum speed during 5‐minute sub‐intervals (average maximum speed) (Figure 2d,f). A one‐way ANOVA of maximum speeds presented statistical significance in the two highest doses (Figure 2g‐j) at two different TOIs (10–35 min and 35‐60 min). Stark differences appear when comparing both methods, as averaging maximum speed over 5‐minute intervals is influenced by long periods of inactivity, hindering the movement amplitude measurement purpose of this metric. Therefore, absolute maximum speed is the preferred method (Figure 2c,e). For haloperidol, only one group (0.5 mg/kg) was significantly different from the saline control between 10 and 35 minutes (Figure 2g). Observingly, the second TOI (35‐60 min) revealed a strong dose dependency for haloperidol (Figure 2h *)*. SCH23390 did not show major differences between 10–35 and 35–60 minutes. Doses of SCH23390 above 0,05 mg/kg were statistically significant in the one‐way ANOVA regardless of the time point measured (Figure 2i‐j).

### 3.3 Dopamine receptor antagonism elicits dose‐dependent akinetic phenotype measured as immobility

**FIGURE 3:**
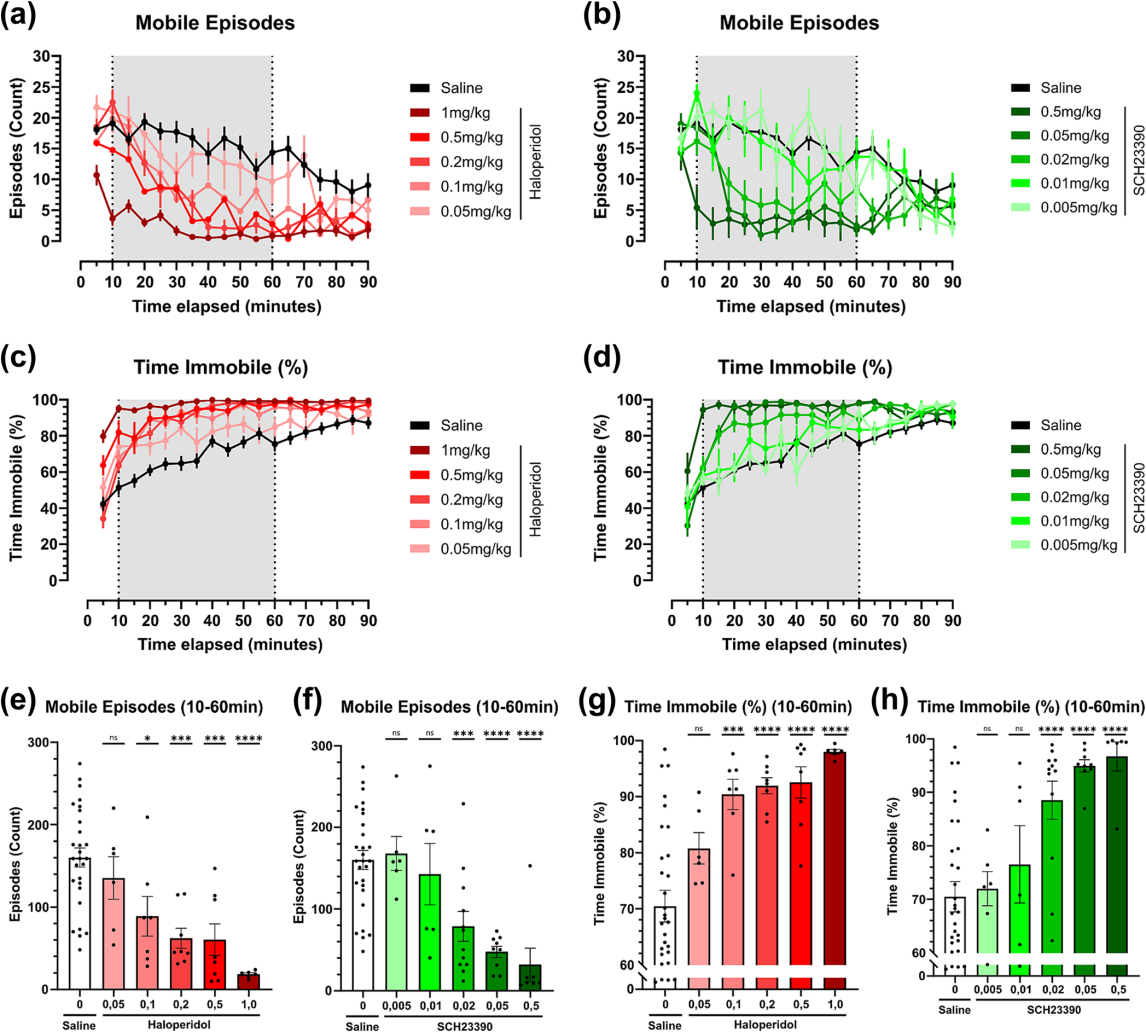
Mobile episode and time immobile metrics of dopamine D2 receptor antagonist haloperidol and D1 receptor antagonist SCH23390 in an open‐field test across an array of five selected doses. (a‐b) Mobile episode count over time in five‐minute intervals of haloperidol (a), corresponding to doses of 1 mg/kg (n = 6), 0.5 mg/kg (n = 8), 0.2 mg/kg (n = 8), 0.1 mg/kg (n = 7) and 0.05 mg/kg (n = 6), and SCH23390 (b), corresponding to doses of 0.5 mg/kg (n = 7), 0.05 mg/kg (n = 9), 0.02 mg/kg (n = 12), 0.01 mg/kg (n = 6) and 0.005 mg/kg (n = 6) compared against a saline control (n = 28). (c‐d) Time spent immobile as a proportion of total time in five‐minute intervals of selected doses of D2R antagonist haloperidol (c) and D1R antagonist SCH23390 (d), compared against a saline control. (e‐f) Mobile episodes count within the TOI comprised between 10‐ and 60 minutes post‐injection of dopamine antagonist haloperidol (e) (F [5, 57] = 10.77) and SCH23390 (f) (F [5, 62] = 10.26). Antagonists are compared through the stipulated dose range compared against a saline control. (g‐h) Proportion of total time immobile within the TOI comprised between 10‐ and 60‐minutes post‐injection of dopamine antagonist haloperidol (g) (F [5, 58] = 11.40) and SCH23390 (h) (F [5, 62] = 9.046). Antagonists are compared through the stipulated dose range compared against a saline control. Brackets above the column graphs denote a one‐way ANOVA analysis, followed by Dunnett's multiple pairwise comparison of the means. NS indicates *p* > 0.05, * *p* < 0.05; ** *p* < 0.01; *** *p* < 0.001; **** *p* < 0.0001. Error bars represent ±SEM.

Next, we evaluated the akinetic behaviour of the mice using mobile episodes and time immobile. The saline group showed a steady decrease in mobile episodes over time. Low antagonist doses showed a similar decreasing pattern, while high doses showed an abrupt post‐injection decrease a few minutes post‐injection (Figure 3a‐b). A 2‐way ANOVA with a Sidak post‐hoc analysis to compare the mobile episode change over time confirms this trend (Tables 1 & 2). Dose dependency appears robust within the TOI, as shown in statistical differences between increasingly higher doses in the one‐way ANOVA (Figure 3e‐f). An alternative to measuring immobile behaviour is to track the proportion of time spent in an immobile state. We chose to represent this metric as a percentage of the total time (Figure 3c‐d, g‐h). High antagonist doses showed a noticeable post‐injection increase, reaching close to 100% immobility within 10 minutes (Figure 3c‐d). A 2‐way ANOVA with a Sidak post‐hoc analysis to compare the time immobile over time confirms this trend (Tables 1 & 2). Time immobile metrics were confined within a small window, as the baseline value for the control group amounted to 70%, reaching over 80% during the last half an hour (Figure 3c‐d). When evaluating the TOI, higher doses of both antagonists translated into larger statistical differences to the saline group in the one‐way ANOVA (Figure 3g‐h).

### 3.4 D1 and D2 receptor antagonism evokes freezing behaviour

**FIGURE 4:**
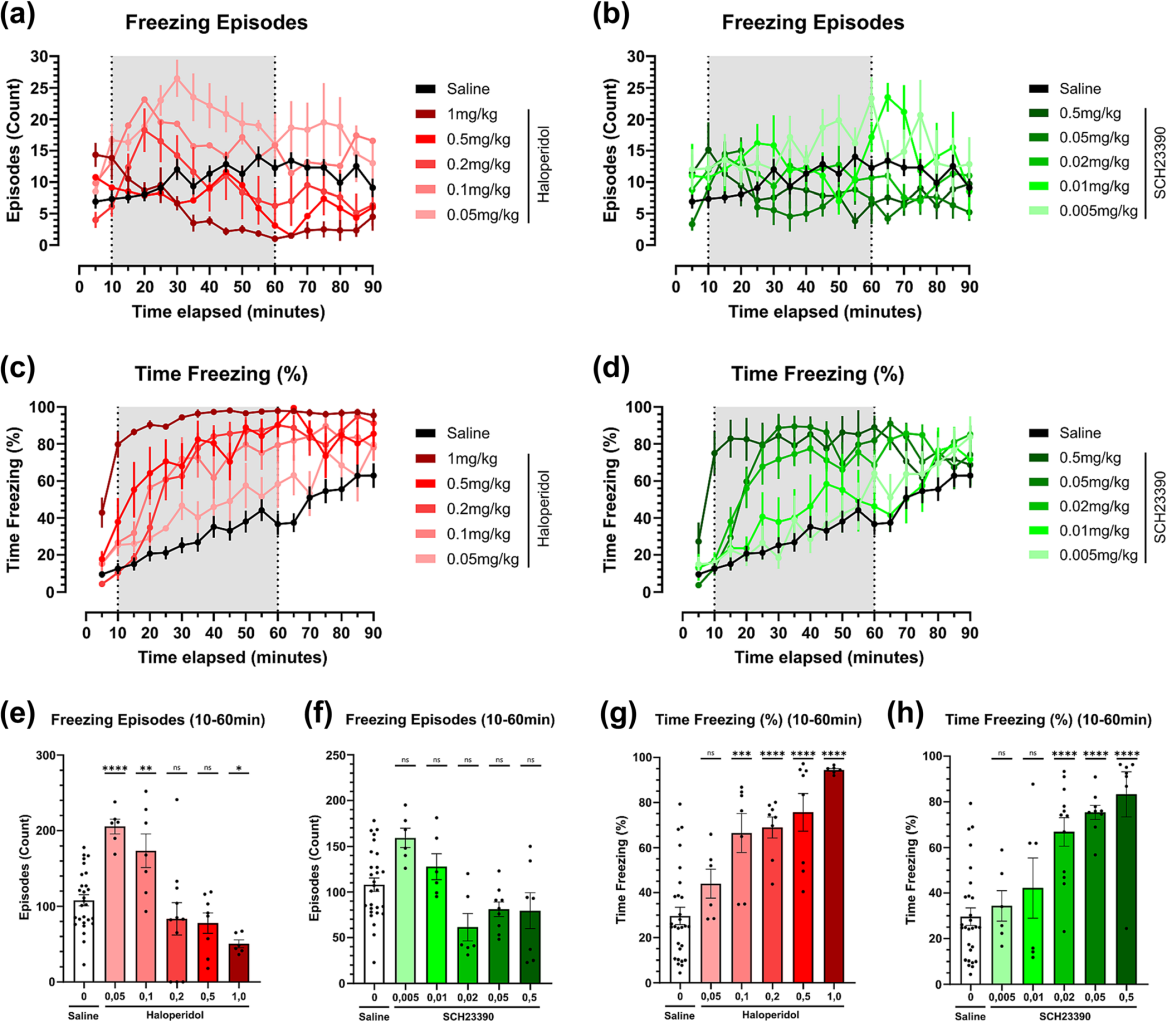
Freezing episodes and time freezing metrics of dopamine D2 receptor antagonist haloperidol and D1 receptor antagonist SCH23390 in an open‐field test across an array of five selected doses. (a‐b) Freezing episode count over time in five‐minute intervals of haloperidol (a), corresponding to doses of 1 mg/kg (n = 6), 0.5 mg/kg (n = 8), 0.2 mg/kg (n = 8), 0.1 mg/kg (n = 7) and 0.05 mg/kg (n = 6), and SCH23390 (b), corresponding to doses of 0.5 mg/kg (n = 7), 0.05 mg/kg (n = 9), 0.02 mg/kg (n = 12), 0.01 mg/kg (n = 6) and 0.005 mg/kg (n = 6) compared against a saline control (n = 28). (c‐d) Time spent freezing as a proportion of total time in five‐minute intervals of selected doses of D2R antagonist haloperidol (c) and D1R antagonist SCH23390 (d), compared against a saline control. (e‐f) Freezing episodes count within the TOI comprised between 10‐ and 60‐minutes post‐injection of dopamine antagonist haloperidol (e) (F [5, 60] = 11.54) and SCH23390 (f)(F [5, 56] = 6.068). Antagonists are compared through the stipulated dose range compared against a saline control. (g‐h) Proportion of total time freezing within the TOI comprised between 10‐ and 60‐minutes post‐injection of dopamine antagonist haloperidol (g) (F [5, 57] = 18.46) and SCH23390 (h) (F [5, 62] = 13.60). Antagonists are compared through the stipulated dose range compared against a saline control. Brackets above the column graphs denote a one‐way ANOVA analysis, followed by Dunnett's multiple pairwise comparison of the means. NS indicates *p* > 0.05, * *p* < 0.05; ** *p* < 0.01; *** *p* < 0.001; **** *p* < 0.0001. Error bars represent ±SEM.

We evaluated freezing behaviour in a similar fashion as immobility. The total number of freezing episodes ‐ the period count in which the animals entered a freezing state, provided a poor locomotor output metric (Figure 4a‐b,e‐f). After injection, freezing episode counts remained constant over time for the saline control group, while increasing at low antagonist doses. A 2‐way ANOVA with a Sidak post‐hoc analysis, evaluating freezing episodes over time shows erratic values, with no clear significance over a reasonably long timeframe (Tables 1 & 2). By analysing the TOI (10 to 60 minutes) using one‐way ANOVA, low antagonist doses elicited a higher number of freezing episodes compared to the control, while high doses elicited the opposite (Figure 4e‐f). Pairwise comparison statistical differences do not show a clear dose‐dependent pattern.

As opposed to freezing episodes, measuring freezing as a proportion of time revealed dose‐dependent effects. Time freezing showed a steady increase in the time proportion in that state in the saline group (Figure 4c‐d). A 2‐way ANOVA with a Sidak post‐hoc analysis to compare the time freezing shows results in line with the pharmacokinetic profile of the compounds (Tables 1 & 2). When evaluating the TOI, dose dependency is strong for haloperidol and SCH23390; stark differences emerged at doses above 0.01 mg/kg haloperidol and 0.02 mg/kg SCH23390 (Figure 4c‐d *)*. This exhibited a similar pattern as observed in time immobile, where higher doses of antagonist yielded a higher proportion of akinesia (Figure 3). When evaluating the TOI using one‐way ANOVA, strong differences emerged at doses starting from 0.1 mg/kg haloperidol and 0.02 mg/kg SCH23390, respectively (Figure 4g‐h).

### 3.5 Animal placement in the open field is not affected by dopamine receptor antagonism

**FIGURE 5:**
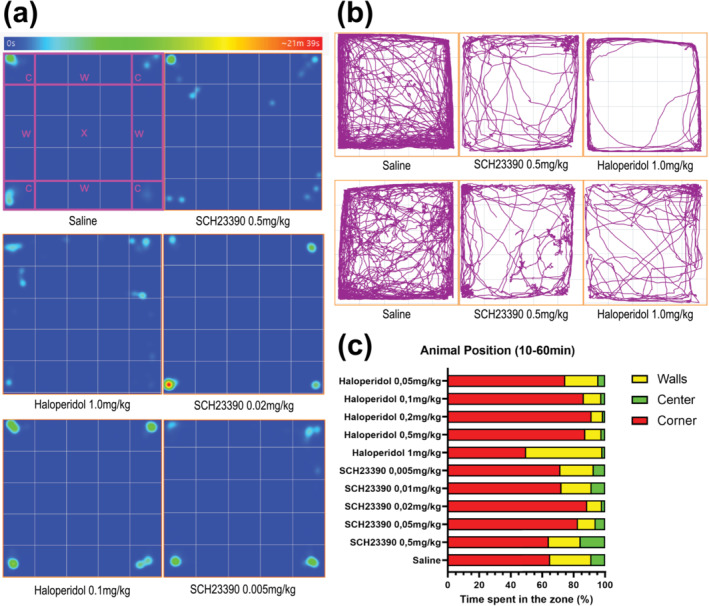
Effect of dopamine antagonism on animal placement behaviour in the open field maze. (a) Position heatmap obtained from averaging all individual animal heatmaps within each dose group. The open field was subdivided into 25 10x10cm segments. The segments were grouped into three zones, delimited in the figure: corner (c), walls (w) and centre (x). Average heatmaps for the saline control (n = 28), haloperidol 1.0 mg/kg (n = 6) and 0,1 mg/kg (n = 7), SCH23390 0.5 mg/kg (n = 7), 0.02 mg/kg (n = 12) and 0.005 mg/kg (n = 6) (b) individual animals track plots of six different animals. Two were administered a saline injection, two were administered a 0.5 mg/kg SCH23390 injection and two were administered a 1.0 mg/kg haloperidol injection. (c) Distribution of time spent in each of the three zones (walls in yellow, centre in green, corner in red) as a proportion of the total time.

**FIGURE 6:**
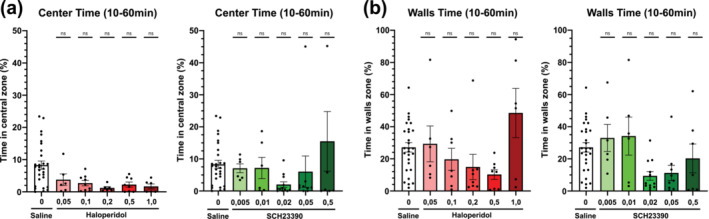
Proportion of time spent in different zones of the open‐field maze, as defined in Figure 5a. (a‐b) Proportion of time spent in the central zone of the open‐field of animals treated with selected doses of dopamine antagonist haloperidol (a) and SCH23390 (b), compared against a saline control. (c‐d) Proportion of time spent in the wall zone of the open‐field of animals treated with selected doses of dopamine receptor antagonist haloperidol (c) and SCH23390 (d), compared against a saline control. Brackets above the column graphs denote a one‐way ANOVA analysis, followed by Dunnett's multiple pairwise comparison of the means. NS indicates *p* > 0.05, * *p* < 0.05; ** *p* < 0.01; *** *p* < 0.001; **** *p* < 0.0001. Error bars represent ±SEM.

Next, we evaluated whether dopamine antagonism affected the spatial behaviour of the mice. We subdivided the open‐field area into 5 × 5 squares, measuring 10x10cm each. The four squares located in the corners were grouped together as the “Corner Zone” (Figure 5a *, Saline, “c”*). The four groups of three squares, centrally located at the sides of the open field, were grouped as the “Walls Zone” (Figure 5a *, Saline, “w”*). The remaining nine squares located in the middle of the arena were grouped as the “Center Zone” (Figure 5a *, Saline, “x”*). Animals tend to spend most of the time in the corner, due to open space‐related anxiety. Centre‐periphery distribution is an anxiety measurement previously used in the literature (Seibenhener & Wooten, 2015). Thus, we conclude that the freezing effect is a locomotor effect and not a consequence of dose‐responsive anxiety. Indeed, by taking a closer look at the animal placement heatmap (Figure 5a), the animal placement was concentrated in the corner zones. Anxiety in open‐field tests has been sufficiently characterized in the literature (Seibenhener & Wooten, 2015). Here, we will focus on the effect of dopamine antagonism on open‐field animal placement. No notable differences between different compounds or dose groups could be drawn from position heatmaps. The track plots represented individual animal trajectories in the open‐field arena (Figure 5b). Individuals treated with high doses of dopamine antagonist showed large qualitative differences in locomotor output compared to the saline control group. Animals treated with high dopamine antagonist doses travelled less through different zones, especially through the central zone. The overall distance travelled was qualitatively reduced compared to the saline group (Figure 5b). We compared the proportions of time spent in each zone to quantitatively compare animal position among the different compound dose repertoires (Figure 5c). Overall, the animals that received SCH23390 or saline spent more time in the wall zone than those that received haloperidol. However, there were no statistical differences in the time spent in the centre or wall zones between any of the dose groups. This was mainly due to the high spread of individual behaviour within dose groups (Figure 6a‐d). Overall, dopamine antagonism did not show any significant differences in spatial animal behaviour.

## 4 DISCUSSION

We found that acute administration of dopamine D1 receptor (SCH23390) and D2 receptor (haloperidol) antagonists in mice induces similar dose‐dependent anti‐kinetic motor effects in the open‐field maze. Our comprehensive depiction of dose–response demonstrates that dopamine antagonism can be used to evoke acute, subtle to intermediate parkinsonian motor phenotypes. We propose that the pharmacological model presented in this study is a feasible model to combine with brain circuit manipulations.

We used the open‐field test, a well‐established tool for assessing spontaneous motor behaviour as well as fear‐ and anxiety‐induced behaviours (Hille et al., 2001; Kraeuter et al., 2019; Seibenhener & Wooten, 2015). This platform was chosen to evaluate parkinsonian motor phenotypes because it allows a comprehensive assessment of various motor outputs within a single session. Platform validation was performed prior to further data analysis. Quadrant placement did not significantly influence motor metrics, while successive open‐field exposure did influence motor metrics. Early open‐field testing rounds presented a higher locomotor output than later rounds (habituation). The design considered these potential confounding effects; as groups had comparable average open‐field test round and quadrant distribution, therefore confirming that these confounding factors did not have an effect on motor measurements. Metrics, such as distance travelled and the number of mobile episodes, are accepted indicators of motor function (Alcacer et al., 2017; Avila‐Luna et al., 2018; Hjorth & Carlsson, 1988; Löschmann et al., 1992; Masini & Kiehn, 2022; Schulz et al., 1985). We focused on distance travelled to gauge overall mobility and mobile episodes to capture spontaneous initiation of locomotor activity. Our data demonstrates robust, dose‐dependent reductions in locomotor activity for both antagonists, confirming their similar effects on motor behaviour and corroborating the notion that this pharmacological approach provides a reliable framework for capturing parkinsonian motor deficits.

We also assessed maximum speed as a metric for parkinsonian bradykinesia. Bradykinesia is characterized by decreased movement amplitude and speed. While these two motor characteristics can be altered by independent mechanisms, the impairment of movement speed is an important characteristic of parkinsonism (Espay et al., 2009). Nevertheless, careful data processing should be performed to extract the correct biological conclusions.

Immobility metrics such as time immobile and the number of mobile episodes provide a value insight into movement initiation. Difficulty initiating movement is a cardinal motor symptom of PD, and the current pharmacological model successfully replicated such characteristics. Time immobile increased with higher doses of antagonist while number of mobile episodes decreased in resemblance with PD characteristics in humans.

Time freezing reliably depicted the akinetic pattern for the entire dose ranges of SCH23390 and haloperidol. Even though some studies have shown that the D2R antagonist haloperidol increased muscle rigidity (Lorenc‐Koci et al., 1996), anxiety and fear (Brandão et al., 2015), we did not observe a significant increase in freezing behaviour relative to the overall distance travelled compared to the groups treated with the D1R antagonist SCH23390. SCH23390 elicited a cleaner akinetic phenotype, where locomotor output was decreased with no perceptible changes in neither muscle rigidity nor overall behaviour. Conversely, the D2‐antagonist haloperidol administration translated to changes in mouse posture, exhibited by an arched back with a hunched tail, lasting even after mobile episodes.

We did not apply specific tests to assess catalepsy, defined as state of sustained immobility and muscle rigidity, where the body or limbs can remain in fixed postures for long periods. Catalepsy can be induced by the D2‐receptor antagonist haloperidol but is not commonly seen in Parkinson's disease. Yet, the rigid posture and resistance to movement seen in catalepsy can resemble the muscular stiffness in Parkinson's disease. Catalepsy assessments, such as the horizontal bar test relies on artificially imposed postures, which cannot be implemented in the open field due to the nature of the platform. Still, it would have been valuable to specifically assess dose‐dependent effects on catalepsy in our study.

We were not able to discern dose‐dependent differences in the centre to peripheral behaviour. The animals did not show a dose‐dependent preference for either corner or wall zones. Centre‐periphery distribution is an anxiety measurement previously used in the literature (Seibenhener & Wooten, 2015). Thus, we conclude that the freezing effect is a locomotor effect and not a consequence of dose‐responsive anxiety. Our data demonstrate that both compounds were able to reliably evoke hypokinetic states. For circuit manipulation experiments, we advise using intermediate doses eliciting moderate motor effects to avoid complete suppression of dopamine transmission. For SCH23390, we recommend a dose of 0.02 mg/kg, while for haloperidol we recommend doses between 0.2 and 0.5 mg/kg. Our results confirm the akinetic phenotype observed in early studies, with distinct qualitative and quantitative differences in maximum speed metrics and motor phenotypes of haloperidol and SCH23390. Several metrics were successfully proven to depict relevant characteristics of parkinsonism, such as bradykinesia, movement initiation and overall locomotor impairment.

Pharmacological models are fast‐acting, transient models for PD research, able to mimic motor deficits derived from dopamine depletion in the striatum (Acuña‐Lizama et al., 2013; Invernizzi & Samanin, 2003; Waku et al., 2021). The major drawback of drug‐based models is the inability to account for the progressive nigrostriatal neurodegeneration and α‐synuclein pathology, limiting their construct validity. Striatal projection neurons expressing D1 and D2 receptors segregate into two distinct circuits, dSPNs and iSPNs that according to the classic model bi‐directionally control movement. The constructive validity of SCH23390 relies on its ability to inhibit dSPN activity and thereby mimicking dopamine depletion of dSPNs (Hjorth & Carlsson, 1988; Schulz et al., 1985; Undie & Friedman, 1988). The effect of SCH23390 antagonism can be predicted by the classical model because dopamine D1 receptors are exclusively expressed on the postsynaptic striatal neurons. Conversely, the expression of dopamine D2 receptors is not limited to iSPNs. The activation of presynaptic D2‐class auto‐receptors in nigrostriatal presynaptic terminals generally causes a decrease in dopamine synthesis, release and firing rates, resulting in hindered locomotor activity, whereas activation of postsynaptic receptors stimulates locomotion. D2‐class auto‐receptors are generally stimulated by a lower concentration of dopamine agonist than postsynaptic receptors. Hence, a dopamine D2R‐agonist can induce a biphasic effect that leads to decreased motor activation at low doses and an increase in locomotion at high doses (Beaulieu & Gainetdinov, 2011). The distribution of D2‐receptors in the striatum has implications for the motor effects of D2R‐antagonists. Acute motor phenotypes induced by D2R‐antagonists are more difficult to explain by the classic model and are more complex due to the pre‐ and post‐synaptic distribution of D2 receptors.

The classic model has also been challenged by an in vivo study in mice which measured selectively the activity of direct‐ and indirect‐pathway spiny projection neurons in the striatum of mice (Cui et al., 2013). Both pathways exhibited transient increases in neural activity when actions are initiated, rather than during inactivity, suggesting that concurrent activation of both pathways may play a crucial role in initiating movement and could have implications for understanding motor symptoms in Parkinson's disease (Cui et al., 2013). Nonetheless, the effect of haloperidol on locomotion is commonly regarded as hypokinetic and cataleptic (Bateup et al., 2010; De Ryck et al., 1980; Ezrin‐Waters & Seeman, 1977; Lapin & Rogawski, 1995; Morelli & Di Chiara, 1985; Sippy & Tritsch, 2023). It also needs to be born in mind that the systemic administration of dopamine receptor antagonists not only affects motor pathways but the entire set of dopamine receptors in the brain (Inoue et al., 1985). Off‐target effects, while not evaluated in this study, can affect murine behaviour in different experimental setups.

Predictive validity of haloperidol‐based models has extensively been proven thanks to the successful recovery of motor impairment with already established PD treatments, such as L‐DOPA and monoamine oxidase B (MAO‐B) inhibitors (Duty & Jenner, 2011). SCH23390 predictive validity has been tested to a much lesser extent. Additionally, a study showed that the D1R agonist SKF38393 used as a substitute for L‐DOPA treatment can prevent motor impairments induced by SCH23390 (Avila‐Luna et al., 2018). Fibre photometry recordings from dSPNs and iSPNs in combination with pharmacological modulation using SCH23390 and the D2R antagonist raclopride, allow monitoring striatal D1/D2 neuron ensemble dynamics at high temporal resolution (Maltese et al., 2021). Another study used knockout mouse lines in combination with haloperidol and L‐DOPA to corroborate the distinct role of dSPNs and iSPNs (Bateup et al., 2010). The use of dopamine antagonists can also be combined with other ligands. For example, microinjection of the GABAergic antagonist picrotoxin in the subthalamic nucleus was shown to elicit motor deficits comparable to haloperidol antagonism (Scheel‐Krüger & Magelund, 1981).

In conclusion, the proposed pharmacological models provide a highly flexible framework for pharmacological studies of acute parkinsonian phenotypes in mice. They do not require specific mouse strains or extensive histological validations. One inherent limitation is the transient nature of the intervention that substantially differs from the slowly progressive neurodegenerative process during PD. Furthermore, the pharmacological models cannot be used for dyskinetic mouse models in which chronic dopamine deprivation and dopamine replacement therapy are a requirement. Despite their inability to model cellular pathophysiology, the pharmacological models portray parkinsonian motor features within a short time frame (Andreoli et al., 2021; Henrich et al., 2018; Masini & Kiehn, 2022). The added flexibility, transient modulation and ease of implementation of pharmacological models can facilitate parallel circuit manipulation studies. Techniques such as chemo−/optogenetics, fibre photometry and electrophysiology in conjunction with pharmacological models can be used to unravel circuit engagement in parkinsonism (Alcacer et al., 2017; Masini & Kiehn, 2022; Zhang et al., 2021).

## AUTHOR CONTRIBUTIONS

**Christian del Agua Villa (CAV):** investigation, formal analysis, data curation, visualization, writing—original draft, writing—review and editing. **Mihai Atudorei (MA):** investigation, formal analysis, data curation, visualization, writing—original draft, writing—review and editing, **Hartwig Roman Siebner (HS):** conceptualization, funding acquisition, project administration, writing—original draft, writing—review and editing, resources. **Mattias Rickhag (MR):** conceptualization, funding acquisition, project administration, supervision, writing—original draft, writing—review and editing, resources.

## CONFLICT OF INTEREST STATEMENT

Hartwig R. Siebner has received honoraria as speaker and consultant from Lundbeck AS, Denmark, and as editor (Neuroimage Clinical) from Elsevier Publishers, Amsterdam, The Netherlands. He has received royalties as book editor from Springer Publishers, Stuttgart, Germany, Oxford University Press, Oxford, UK, and from Gyldendal Publishers, Copenhagen, Denmark.

### PEER REVIEW

The peer review history for this article is available at https://www.webofscience.com/api/gateway/wos/peer‐review/10.1111/ejn.16622.

## Data Availability

Raw data will be made publicly available using a public access repository.
